# Isolation of *Staphylococcus aureus* and Antibiotic-Resistant *Staphylococcus aureus* from Residential Indoor Bioaerosols

**DOI:** 10.1289/ehp.9585

**Published:** 2006-09-07

**Authors:** Angelina Gandara, Linda C. Mota, Carissa Flores, Hernando R. Perez, Christopher F. Green, Shawn G. Gibbs

**Affiliations:** 1 The University of Texas Health Science Center at Houston, School of Public Health, El Paso, Texas, USA; 2 Drexel University, School of Public Health, Philadelphia, Pennsylvania, USA; 3 Department of Civil and Environmental Engineering, University of Cincinnati, Cincinnati, Ohio, USA

**Keywords:** antibiotic resistant, bioaerosols, residential indoor air, *Staphylococcus aureus*

## Abstract

**Objective:**

In this study we evaluated the levels of *Staphylococcus aureus* and antibiotic-resistant *S. aureus* in colony-forming units (CFU) per cubic meter of air.

**Design:**

We used Andersen two-stage samplers to collect bioaerosol samples from 24 houses in El Paso, Texas, using tryptic soy agar as the collection media, followed by the replicate plate method on Chapman Stone selective medium to isolate *S. aureus.* The Kirby-Bauer disk diffusion method was used to determine antibiotic resistance to ampicillin, penicillin, and cefaclor, which represent two distinct classes of antibiotics.

**Results:**

The average recovered concentration of respirable heterotrophic organisms found outside each home was 345.38 CFU/m^3^, with an average of 12.63 CFU/m^3^ for *S. aureus*. The average recovered concentration of respirable heterotrophic organisms found inside each home was 460.23 CFU/m^3^*,* with an average of 15.39 CFU/m^3^ for *S. aureus.* The respirable *S. aureus* recovered from inside each home had an average resistance of 54.59% to ampicillin and 60.46%. to penicillin. Presence of cefaclor-resistant and of multidrug-resistant *S. aureus* was the same, averaging 13.20% per house. The respirable *S. aureus* recovered from outside each home had an average resistance of 34.42% to ampicillin and 41.81% to penicillin. Presence of cefaclor-resistant and of multidrug-resistant *S. aureus* was the same, averaging 13.96% per house.

**Conclusions:**

This study indicates that antibiotic-resistant bioaerosols are commonly found within residential homes. Our results also suggest that resistant strains of airborne culturable *S. aureus* are present in higher concentrations inside the study homes than outside the homes.

The introduction of antimicrobial agents to treat human disease was one of the most significant public health accomplishments of the 20th century. Although many factors (e.g., improvements in sanitation, nutrition, and standard of living) worked in concert with antibiotic therapy to control and limit infectious disease transmission, antibiotic therapy was unique in that it not only allowed for the prevention but also for the curing and control of certain diseases (Barker 1999; Cohen 1992). Although the introduction and successful development of this therapeutic class of agents represents a significant medical achievement, this success has also led to complacency within both greater society and the scientific community with regard to the development of bacterial resistance (Neu 1992). That micro-organisms possess the ability to develop resistance to antibiotics was recognized soon after the introduction of antimicrobial therapy, when resistant bacteria were initially identified in the 1930s (Smith and Coast 2002; Virk and Steckelberg 2000). Reoccurring episodes of infection with multiple resistant organisms have affected hospitals since the early 1950s when penicillin-resistant staphylococci initially emerged (Cohen 1992). In spite of the increased understanding of the factors contributing to the development of resistance over the last 60 years, the extent of this problem has not decreased with time and is currently among the strongest global threats to the treatment of infectious disease (Conly 2002). The degree to which this problem has progressed is demonstrated by the fact that resistance has developed against all available classes of antibiotics (Rao 1998; Virk and Steckelberg 2000).

As a result of the significant difficulties associated with the accurate measurement of airborne bacteria, little data exist on the concentration of bacteria in indoor air in comparison to other airborne contaminants [Institute of Medicine Committee on Damp Indoor Spaces and Health (IOM) 2004]. Although there are significant difficulties in measuring airborne concentrations of most bioaerosols, the health effects associated with exposure to aerosolized bacteria have received less research attention than those associated with exposure to other organic dusts, such as molds and animal allergens. Also, many bacteria have traditionally been thought of as infectious organisms, with disease resulting only from transmission via large droplets over short distances or through contact with contaminated surfaces (Roy and Milton 2004). Roy and Milton (2004) suggested that this paradigm be questioned and that there is a need for improved understanding of aerosol-acquired disease. This need is made more urgent by the increasing environmental burden of antibiotic-resistant bacteria.

Multidrug-resistant *Staphylococcus aureus* is an example of a bacterium for which the role of exposure to aerosolized organisms in disease transmission should be more closely evaluated. An international survey of infections due to *Staphylococcus* species resulted in the finding that *S. aureus* was the most prevalent cause of hospital- and community-acquired bloodstream, skin and soft tissue, and lower respiratory infection (Diekema et al. 2001). In the hospital setting, the most common mode of transmission of resistant *S. aureus* is close contact with infected persons or with health-care workers with contaminated hands or clothing (Cooper et al. 2004). Recent evidence suggests, however, that airborne dispersal and transmission may also be important (Beggs 2003; Cooper et al. 2004; Roberts et al. 2006), and case studies implicating airborne transmission in the hospital setting have been published in the literature (Cotterill et al. 1996; Wagenvoort et al. 1993). Although drug-resistant *S. aureus* has historically been a significant problem only in hospitals, the urgent need for further study of the ambient airborne concentrations and the role of airborne transmission of this organism in non-hospital environments is demonstrated by the increasing prevalence of methicillin-resistant *S. aureus* (MRSA) infections in the community (Chambers 2001). Most alarming about this trend is that infection has been observed among individuals with and without known risk factors (Gorak et al. 1999).

The primary objective of this study was to determine the levels of respirable *S. aureus*, including antibiotic-resistant and multidrug-resistant *S. aureus* (those resistant to at least two classes of antibiotics) found within the bio-aerosols of residential homes. We hypothesized that *S. aureus*, including antibiotic-resistant and multidrug-resistant *S. aureus*, were present in the bioaerosols of the average home.

## Methods

### Sample collection

Indoor and outdoor culturable bacterial bioaerosol samples were collected in El Paso, Texas, from 24 one-story houses that had no basement or attic. These 24 houses were randomly selected from a separate larger study of 50 houses that had been identified when a solicitation for study participation was sent to faculty, staff, and students at the University of Texas School of Public Health, El Paso Regional Campus, and the University of Texas at El Paso (Mota LC, Gibbs SG, Green CF, Payan F, Tarwater PM, Ortiz M, unpublished data). These houses contained no visible microbial issues; no adverse health issues that could be related to microbial contamination of the home had been previously reported by the occupants. Protocols were in accordance with Human Subject and Health Insurance Portability and Accountability Act approval through the University of Texas School of Public Health. Samples were taken during spring (March, April, and May) of 2006, with sampling conducted at different times of day to accommodate the needs of the home owners.

We used methods adapted from previous work (Gibbs et al. 2004, 2006; Green et al. 2003; Mota LC, Gibbs SG, Green CF, Payan F, Tarwater PM, Ortiz M, unpublished data). Duplicate bioaerosol samples were collected inside each house using Andersen two-stage viable microbial particle sizing sampler instruments (Tisch Environmental, Cleves, OH) (Andersen Samplers Inc. 1976.) The Andersen two-stage sampler is a cascade impactor with 200 orifices for each of the two stages; the sampler separates particles according to their size onto one of two stages. The nonrespirable stage collects particles > 8 μm onto a media-filled petri dish, and the respirable stage collects particles of 0.8–8 μm onto a second media-filled petri dish. Separate equipment, including a pump (Gast Oil-less Pressure/Vacuum Pump, Gast Manufacturing, Inc., Benton Harbor, MI) and Andersen two-stage sampler were used to duplicate each site. All pumps were calibrated to 28.3 L/min using the TriCal Laboratory/Field Audit Calibrator (BGI Incorporated, Waltham, MA) before each sampling event (Dacarro et al. 2003). The samplers were placed on a tripod 1 m above the floor surface in the center of the main living area to simulate the human breathing zone (Hyvarinen et al. 2001; Pastuska et al. 2000; Sterling and Lewis 1998).

The Andersen two-stage samplers were loaded with plates of tryptic soy agar (TSA; Difco Laboratories, Detroit, MI), an excellent general agar used to culture a variety of bacterial microorganisms. Duplicate 10- and 15-min air samples were taken at each home. Various climatic conditions were measured, including temperature, relative humidity, and barometric pressure, using a portable weather station (Traceable Digital Barometer Module; Calibration Control Company, Friendswood, TX). All equipment and materials were handled using aseptic technique to ensure that the bioaerosol samples were not contaminated. All samples were returned to the laboratory for analysis within 2 hr of sample collection.

At the laboratory, the plates were placed in an inverted position in an incubator at 35°C. The colonies were counted after 24 and 48 hr to determine if the plates were overgrown. After 48 hr of incubation, the plates were inverted and refrigerated at 4°C until use for the replica plate method (Lederberg and Lederberg 1952). We counted colonies for each plate and calculated the total number of culturable colony forming units (CFUs) per cubic meter (Green et al. 2003; Meklin et al. 2002). Only the TSA plates from the respirable portion were evaluated using the replica plate method and Kirby-Bauer procedures; therefore, the data presented in the present study deal only with the culturable respirable portion of bioaerosols.

### Isolation and speciation

We used the replica plate method to identify the recovered aerosolized culturable bacteria by transferring the bacterial colonies onto a selective medium (Lederberg and Lederberg 1952). For this method we used Chapman Stone medium (CSM; Difco Laboratories) for identification of *S. aureus*. After pressing of the CSM, TSA was used as a final control for the method, being pressed first and last to ensure that the organisms were completely transferred to all plates. All plates were incubated at 35°C and counted at 24 and 48 hr. The presence of *S. aureus* was further confirmed using Bacto coagulase plasma (Fisher Scientific, Houston, TX). Once counted, the plates were refrigerated in an inverted position at 4°C until used for the Kirby-Bauer disk diffusion method.

### Antimicrobial susceptibility testing

We analyzed the antibiotic-resistant characteristics of up to five *S. aureus* isolates collected from the respirable plate of each sample using the Kirby-Bauer disk diffusion method (Bauer et al. 1966). Only organisms that could be isolated from the CSM and cultured using a slant tube were evaluated for antibiotic resistance. The method was conducted in duplicate for each organism evaluated. If the duplicate analysis did not result in the same resistance profile, the organism was not included in the analysis. A sterile loop was used to transfer the micro-organism being tested to a tube of phosphate-buffer water until the tube was the same turbidity as the 0.5 McFarland standard, which resulted in an estimated 10^8^ CFUs/mL. Room temperature Mueller-Hinton agar (MHA; Difco Laboratories) plates were used to evaluate each of the microorganisms for antibiotic resistance. Once the organism was placed onto the MHA, it was allowed to dry before the disks containing the antibiotics were placed onto the MHA surface using a Sensi-Disk 6-Place Self-Tamping Dispenser (Baltimore Biological Laboratory, Cockeysville, Maryland). The disks were allowed to settle before being inverted and placed in an incubator at 35°C. The plates were checked for susceptibility after 48 hr. The zones of inhibition were recorded for all of the plates, and a determination was made as to whether the microorganism was susceptible, intermediately resistant, or resistant to each antibiotic evaluated using National Committee for Clinical Laboratory Standards (NCCLS 1997, 2000, 2001). Table 1 provides the specific NCCLS zone diameters used to categorize *S. aureus.* An organism was not included in the tabulation of results if the antibiotic disk had dislodged from the media or if the duplicates did not result in the same antibiotic-resistance profile.

We used three types of Antibiotic Susceptibility Test Disks (Difco Laboratories) for the Kirby-Bauer method. These three antibiotics (10 μg ampicillin, 10 μg penicillin, and 30 μg cefaclor), chosen because they are commonly used in human medicine, represent two distinct classes of antibiotics. Ampicillin and penicillin are both penicillins, and cefaclor is a second generation cephalosporin. Multidrug resistance is defined as resistance to at least two different classes of antibiotics; therefore, any organism in this study that was resistant to cefaclor and either ampicillin or penicillin was deemed multidrug resistant.

Quality assurance and quality control was maintained throughout the study using aseptic techniques. All sampling material that could be autoclaved was autoclaved for 15 min at 15 psi and 121°C. The Andersen two-stage samplers were sterilized after each use, washed, and then sterilized again before their next use. All other items were disinfected with a 70% ethanol solution after each sampling trip and before the next sampling trip. For negative controls, we used empty plates of TSA that were taken to the sampling site during collection. *S. aureus* (ATCC 25923; American Type Culture Collection, Manassas, VA) was used as positive control. We applied *S. aureus* (ATCC 25923) to the CSM to ensure that it would be able to culture the selected organism. We also tested the control organisms in the Kirby-Bauer method to ensure that antibiotics used would inhibit growth of a nonresistant culture.

## Results

Climatic conditions measured both inside and outside each home during the course of the study showed an expected variation for a study conducted during the spring season (Table 2).

The average recovered concentration of respirable heterotrophic organisms found within each home was 460.23 CFU/m^3^ (Table 3). A quartile (Q) representation shows the distribution of respirable heterotrophic organisms from inside the homes as follows: Q_0_ = 154.59, Q_1_ = 269.29, Q_2_ = 415.49, Q_3_ = 657.69, and Q_4_ = 883.39 CFU/m^3^. The average recovered concentration of *S. aureus* found within each home was 15.39 CFU/m^3^ (Table 3). A quartile representation shows the distribution of respirable *S. aureus* from inside the homes at Q_0_ = 0.59, Q_1_ = 4.20, Q_2_ = 10.31, Q_3_ = 20.32, and Q_4_ = 53.30 CFU/m^3^. This results in an average respirable percentage of recovered culturable *S. aureus* to heterotrophic organisms of 3.94% (Q_0_ = 0.27%, Q_1_ = 1.11%, Q_2_ = 2.37%, Q_3_ = 5.01%, and Q_4_ = 20.10%).

The average recovered concentration of respirable heterotrophic organisms found outside each home was 345.38 CFU/m^3^ (Table 4). The distribution of respirable heterotrophic organisms from outside the homes by quartile were Q_0_ = 44.17, Q_1_ = 153.56, Q_2_ = 286.51, Q_3_ = 441.70, and Q_4_ = 883.39 CFU/m^3^. The average recovered concentration of *S. aureus* found outside each home was 12.63 CFU/m^3^ (Table 4). A quartile representation shows the distribution of respirable *S. aureus* from outside the homes at Q_0_ = 0.00, Q_1_ = 3.61, Q_2_ = 6.77, Q_3_ = 11.34, and Q_4_ = 74.79 CFU/m^3^. This results in an average respirable percentage of recovered culturable *S. aureus* to heterotrophic organisms of 5.08% (Q_0_ = 0.00%, Q_1_ = 1.23%, Q_2_ = 2.44%, Q_3_ = 5.42%, and Q_4_ = 37.33%); indicating that, on average, *S. aureus* represents 5.08% of the recovered culturable outdoor bioaerosol.

The respirable *S. aureus* recovered from inside each home had an average resistance to ampicillin of 54.59% (Q_0_ = 0.00%, Q_1_ = 34.31%, Q_2_ = 61.54%, Q_3_ = 67.95%, and Q_4_ = 100.00%) (Table 5). The average resistance to penicillin was 60.46% for each home (Q_0_ = 0.00%, Q_1_ = 45.00%, Q_2_ = 62.50%, Q_3_ = 75.00%, and Q_4_ = 100.00%). Presence of cefaclor-resistant and of multidrug-resistant *S. aureus* was the same, averaging 13.20% per house (Q_0_ = 0.00%, Q_1_ = 12.50%, Q_2_ = 23.61%, Q_3_ = 60.00%, and Q_4_ = 60.00%).

The average resistance to ampicillin of respirable *S. aureus* recovered from outside each home was 34.42% (Q_0_ = 0.00%, Q_1_ = 0.00%, Q_2_ = 33.33%, Q_3_ = 50.00%, and Q_4_ = 100.00%) (Table 6). The average resistance to penicillin was 41.81% for each home (Q_0_ = 0.00%, Q_1_ = 0.00%, Q_2_ = 40.20%, Q_3_ = 72.50%, and Q_4_ = 100.00%). Presence of cefaclor-resistant and of multidrug-resistant *S. aureus* was the same, averaging 13.96% per house (Q_0_ = 0.00%, Q_1_ = 0.00%, Q_2_ = 0.00%, Q_3_ = 20.83%, and Q_4_ = 100.00%).

Using a paired *t*-test, we found that resistant strains of airborne culturable *S. aureus* were significantly higher inside the study homes than outside the homes. However, we found no significant difference between the concentrations of *S. aureus* inside versus outside the homes.

## Discussion

The present study was conducted over a 3-month period during spring 2006 to test the hypothesis that *S. aureus*, including antibiotic-resistant and multidrug-resistant *S. aureus*, are present in the bioaerosol of the average home. Study findings not only support our hypothesis but also suggest that resistant strains of airborne culturable *S. aureus* are present in higher concentrations inside the study homes than outside of the homes (Tables 5 and 6). This data trend suggests the presence of an indoor source of these organisms. Although this trend may be, in part, the result of the significantly higher concentrations of total heterotrophic organisms recovered inside compared to outside the homes, the lack of significant differences in both concentrations of recovered *S. aureus* and the percentages of *S. aureus* within all heterotrophic organisms recovered suggests that greater numbers of culturable antibiotic-resistant *S. aureus* strains per unit volume of air are present inside the homes. Additionally, based on evidence suggesting that human activities involving the movement of dry fabrics, such as bed making and curtain movement, are associated with elevated concentrations of airborne bacteria (Das et al. 2002; Roberts et al. 2006), it is reasonable to assume that elevated concentrations of *S. aureus* may exist indoors compared to outdoors. The health risks associated with occupant exposure to the concentrations we observed are difficult to calculate because of the few studies that have reported bacterial aerosol concentrations in indoor air (IOM 2004) and the resultant lack of epidemiologic data available for typical indoor environments. Although airborne bacteria have been associated with infectious and allergic respiratory disease (Heidelberg et al. 1997), research involving bioaerosol assessment of culturable organisms in the residential environment has largely focused on fungi and the allergenic and asthmatic health effects associated with exposure to these organisms. The results of the present study, in combination with the recently observed trends in the epidemiology of *S. aureus* (Chambers 2001) provide sufficient justification for further scientific evaluation of both the indoor sources of, and health effects associated with, indoor residential exposure to airborne *S. aureus*.

The epidemiology of *S. aureus* has changed in that there has been a continual increase in the prevalence of infections caused by antibiotic-resistant strains of the microbe (Garcia-Lara et al. 2005; Maranan et al. 1997). In addition to this increase in prevalence, resistant *S. aureus* has progressed from being an organism primarily associated with nosocomial infection to one that has begun to regularly infect individuals outside of the hospital setting (Chambers 2001; Garcia-Lara et al. 2005; Groom et al. 2001; Herold et al. 1998; Manranan et al. 1997; Salgado et al. 2003). The lack of published data documenting patterns in bacterial aerosol concentrations in residential environments over time precludes our ability to determine whether there has been an increase in airborne concentrations of resistant *S. aureus* that has occurred in parallel with the increase in community-acquired infection. However, our finding of elevated indoor concentrations compared to outdoor reference samples indicates the need for further evaluation of the implications of this indoor–outdoor relationship. The extent to which these elevated concentrations increase the risk of adverse health outcomes or simply reflect the increasing burden of resistant bacteria in our environment are issues that merit the attention of the scientific community. This attention is particularly needed for the most vulnerable in society. Much of the concern regarding the emergence of resistant *S. aureus* infections in the community has resulted from reports of methicillin-resistant *S. aureus* (MRSA) isolated from children (Chambers 2001). *S. aureus* is a significant pathogen in children and causes illnesses ranging from minor soft tissue lesions to life-threatening infections (Marcinak and Frank 2003). Risk factors for MRSA infection have traditionally been thought to include intravenous drug use, serious underlying illness, previous antimicrobial therapy, prolonged hospitalization, and invasive or surgical procedures (Herold et al. 1998; Maranan et al. 1997). However, since the 1990s there has been an increase in community-acquired MRSA infections among healthy children without known risk factors [Centers for Disease Control and Prevention (CDC) 2006; Marcinak and Frank 2003]. This increase has been documented in both hospital-based studies (Gorak et al. 1999; Herold et al. 1998; Hussain et al. 2000) and case reports of outbreaks among newborns (CDC 2006), as well as infections resulting in the death of four children in the midwestern United States (CDC 1999).

In any investigation, potential confounders in the role that airborne *S. aureus* plays in adverse human health outcomes include its near ubiquity in the human environment and its carriage by approximately 30% of healthy humans (Garcia-Lara et al. 2005). Rates of infection are higher in carriers than non-carriers, and nasal carriage of the organism is a major risk factor for staphylococcal disease (Peacock et al. 2001). To clearly discern the role of the airborne bacterium in the causation of health effects, most epidemiologic investigations would benefit from separate assessments of carriers and noncarriers. The near ubiquity of the organisms in the human environment presents significant challenges in not only the measurement of airborne concentrations but also in the determination of potential sources of any elevated concentrations. These challenges are present in addition to those already limiting the assessment of bacterial aerosols in the indoor environment. These limitations, while significant, should not prevent future investigation into this important issue.

## Conclusions

Culturable *S. aureus* was recovered inside each of 24 houses sampled and in 23 of the outdoor reference samples. *S. aureus* isolates resistant to both penicillin and ampicillin were recovered inside 22 of 24 study homes, whereas isolates resistant to cefaclor were recovered in 14 of 24 homes. Trends in our data suggest that resistant strains were present in higher concentrations inside homes compared to outside the homes. The findings of the present study, in combination with existing literature documenting the increase in community-acquired infections caused by resistant strains of *S. aureus,* indicate the need for further research into the role that aerosolized organisms play in the causation of adverse health of building occupants.

## Figures and Tables

**Table 1:** NCCLS zone diameters used to categorize *S. aureus* recovered as susceptible, intermediate, or resistant.

		Zone diameter interpretive standards (mm)^a^		
Antimicrobial agent	Disk potency (μg)	Resistant	Intermediate	Susceptible
Ampicillin	10	≤ 28	—	≥ 29
Cefaclor	30	≤ 14	15–17	≥ 18
Penicillin	10	≤ 28	—	≥ 29

a These standards were adapted from NCCLS (1997, 2000, 2001).

**Table 2:** Summary of climatic conditions for all homes.

	Mean ± SD	Minimum	Maximum
Temperature (°C)			
Inside	18.96 ± 2.14	15	24
Outside	15.38 ± 6.01	7	30
Relative humidity (%)			
Inside	27.08 ± 3.96	23	42
Outside	27.50 ± 4.27	20	38
Barometric pressure (mmHg)			
Inside	882.63 ± 7.20	869	895
Outside	882.58 ± 7.08	869	894

**Table 3:** Average recovered concentrations (CFU/m^3^) of respirable *S. aureus* and heterotrophic organisms from inside each home.

House	*S. aureus*	Heterotrophic organisms	*S. aureus* within heterotrophic organisms (%)
1	2.94	173.73	1.69
2	7.36	692.87	1.06
3	4.42	607.18	0.73
4	29.74	389.87	7.63
5	17.08	623.38	2.74
6	9.42	839.22	1.12
7	11.19	505.89	2.21
8	5.01	154.59	3.24
9	9.42	456.12	2.07
10	37.69	883.39	4.27
11	13.55	274.15	4.94
12	20.02	790.64	2.53
13	20.02	673.14	2.97
14	3.53	328.92	1.07
15	13.25	254.71	5.20
16	0.59	209.95	0.28
17	6.48	441.11	1.47
18	1.77	655.48	0.27
19	3.24	214.66	1.51
20	2.65	373.67	0.71
21	39.46	318.61	12.38
22	53.30	664.31	8.02
23	21.20	336.28	6.30
24	35.92	178.74	20.10
Mean	15.39	460.23	3.94
Q_0_	0.59	154.59	0.27
Q_1_	4.20	269.29	1.11
Q_2_	10.31	415.49	2.37
Q_3_	20.32	657.69	5.01
Q_4_	53.30	883.39	20.10

**Table 4:** Average recovered concentrations (CFU/m^3^) of respirable *S. aureus* and heterotrophic organisms from outside each home.

House	*S. aureus*	Heterotrophic organisms	*S. aureus* within heterotrophic organisms (%)
1	4.12	223.20	1.85
2	7.66	159.01	4.81
3	3.83	723.20	0.53
4	0.00	62.43	0.00
5	5.89	213.19	2.76
6	4.12	243.42	1.69
7	24.73	278.56	8.88
8	2.36	137.22	1.72
9	8.83	356.30	2.48
10	5.89	81.27	7.25
11	2.94	242.05	1.22
12	10.60	441.11	2.40
13	9.42	294.46	3.20
14	0.59	108.36	0.54
15	32.98	883.39	3.73
16	2.36	443.46	0.53
17	13.55	402.83	3.36
18	2.36	883.39	0.27
19	6.48	375.74	1.72
20	7.07	574.20	1.23
21	74.79	641.64	11.66
22	45.35	378.09	11.99
23	16.49	44.17	37.33
24	10.60	98.35	10.78
Mean	12.63	345.38	5.08
Q_0_	0.00	44.17	0.00
Q_1_	3.61	153.56	1.23
Q_2_	6.77	286.51	2.44
Q_3_	11.34	441.70	5.42
Q_4_	74.79	883.39	37.33

**Table 5:** Antibiotic resistance of evaluated recoverable respirable *S. aureus* from inside each home.

		Ampicillin		Penicillin		Cefaclor		Multidrug	
House	*N*	*N*_r_	Percent	*N*_r_	Percent	*N*_r_	Percent	*N*_r_	Percent
1	1	0	0.00	0	0.00	0	0.00	0	0.00
2	6	2	33.33	3	50.00	1	16.67	1	16.67
3	7	2	28.57	2	28.57	0	0.00	0	0.00
4	8	6	75.00	6	75.00	2	25.00	2	25.00
5	7	4	57.14	5	71.43	2	28.57	2	28.57
6	8	5	62.50	4	50.00	0	0.00	0	0.00
7	8	2	25.00	3	37.50	1	12.50	1	12.50
8	3	2	66.67	3	100.00	0	0.00	0	0.00
9	4	1	25.00	3	75.00	1	25.00	1	25.00
10	17	6	35.29	6	35.29	1	5.88	1	5.88
11	8	6	75.00	5	62.50	1	12.50	1	12.50
12	9	6	66.67	6	66.67	2	22.22	2	22.22
13	11	7	63.64	7	63.64	0	0.00	0	0.00
14	5	2	40.00	2	40.00	1	20.00	1	20.00
15	16	8	50.00	8	50.00	4	25.00	4	25.00
16^a^	0	—	—	—	—	—	—	—	—
17	5	4	80.00	4	80.00	3	60.00	3	60.00
18	6	3	50.00	3	50.00	0	0.00	0	0.00
19	2	2	100.00	2	100.00	0	0.00	0	0.00
20	3	2	66.67	3	100.00	0	0.00	0	0.00
21	13	9	69.23	9	69.23	1	7.69	1	7.69
22	12	4	33.33	4	33.33	0	0.00	0	0.00
23	13	8	61.54	8	61.54	2	15.38	2	15.38
24	11	10	90.91	10	90.91	3	27.27	3	27.27
Mean			54.59		60.46		13.20		13.20
Q_0_			0.00		0.00		0.00		0.00
Q_1_			34.31		45.00		12.50		12.50
Q_2_			61.54		62.50		23.61		23.61
Q_3_			67.95		75.00		60.00		60.00
Q_4_			100.00		100.00		60.00		60.00

Abbreviations: *N*, number or organisms evaluated; *N*_r_, number of resistant organisms.

a No organisms could be isolated for resistance testing.

**Table 6:** Antibiotic resistance of evaluated recoverable respirable *S. aureus* from outside each home.

		Ampicillin		Penicillin		Cefaclor		Multidrug	
House	*N*	*N*_r_	Percent	*N*_r_	Percent	*N*_r_	Percent	*N*_r_	Percent
1	3	1	33.33	3	100.00	1	33.33	1	33.33
2	2	0	0.00	0	0.00	0	0.00	0	0.00
3	3	1	33.33	1	33.33	1	33.33	1	33.33
4^a^	0	—	—	—	—	—	—	—	—
5	2	0	0.00	0	0.00	0	0.00	0	0.00
6	2	0	0.00	0	0.00	0	0.00	0	0.00
7	10	7	70.00	7	70.00	0	0.00	0	0.00
8	1	0	0.00	0	0.00	0	0.00	0	0.00
9	5	1	20.00	1	20.00	0	0.00	0	0.00
10	1	0	0.00	0	0.00	0	0.00	0	0.00
11^a^	0	—	—	—	—	—	—	—	—
12	6	2	33.33	3	50.00	0	0.00	0	0.00
13	7	5	71.43	6	85.71	0	0.00	0	0.00
14^a^	0	—	—	—	—	—	—	—	—
15	4	1	25.00	1	25.00	0	0.00	0	0.00
16	2	0	0.00	0	0.00	0	0.00	0	0.00
17	5	4	80.00	4	80.00	2	40.00	2	40.00
18	1	1	100.00	1	100.00	1	100.00	1	100.00
19	2	1	50.00	1	50.00	0	0.00	0	0.00
20	2	1	50.00	2	100.00	1	50.00	1	50.00
21	17	8	47.06	8	47.06	1	5.88	1	5.88
22	4	1	25.00	1	25.00	0	0.00	0	0.00
23^a^	0	—	—	—	—	—	—	—	—
24	6	3	50.00	3	50.00	1	16.67	1	16.67
Mean			34.42		41.81		13.96		13.96
Q_0_			0.00		0.00		0.00		0.00
Q_1_			0.00		0.00		0.00		0.00
Q_2_			33.33		40.20		0.00		0.00
Q_3_			50.00		72.50		20.83		20.83
Q_4_			100.00		100.00		100.00		100.00

Abbreviations: *N*, number or organisms evaluated; *N*_r_, number of resistant organisms.

a No organisms could be isolated for resistance testing.
